# Interplay of Kinetic and Thermodynamic Reaction Control Explains Incorporation of Dimethylammonium Iodide into CsPbI_3_

**DOI:** 10.1021/acsenergylett.2c00877

**Published:** 2022-07-26

**Authors:** Aditya Mishra, Dominik J. Kubicki, Ariadni Boziki, Rohit D. Chavan, Mathias Dankl, Marko Mladenović, Daniel Prochowicz, Clare P. Grey, Ursula Rothlisberger, Lyndon Emsley

**Affiliations:** † Laboratory of Magnetic Resonance, Institut des Sciences et Ingénierie Chimiques, École Polytechnique Fédérale de Lausanne (EPFL), 1015 Lausanne, Switzerland; ‡ Department of Physics, University of Warwick, CV4 7AL, Coventry, United Kingdom; § Laboratory of Computational Chemistry and Biochemistry, Institut des Sciences et Ingénierie Chimiques, École Polytechnique Fédérale de Lausanne (EPFL), 1015 Lausanne, Switzerland; ∥ Institute of Physical Chemistry, Polish Academy of Sciences, Kasprzaka 44/52, 01-224 Warsaw, Poland; ⊥ Yusuf Hamied Department of Chemistry, University of Cambridge, Lensfield Road, Cambridge, CB2 1EW, United Kingdom

## Abstract

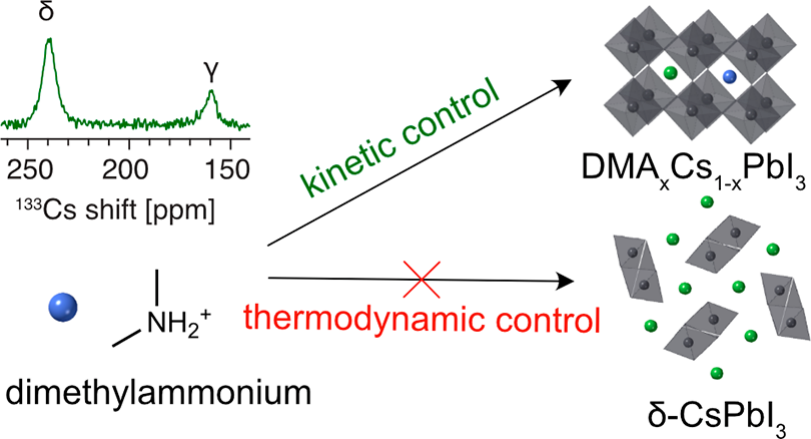

CsPbI_3_ is a promising material for optoelectronics owing to its thermal robustness and favorable bandgap. However, its fabrication is challenging because its photoactive phase is thermodynamically unstable at room temperature. Adding dimethylammonium (DMA) alleviates this instability and is currently understood to result in the formation of DMA_*x*_Cs_1–*x*_PbI_3_ perovskite solid solutions. Here, we use NMR of the ^133^Cs and ^13^C local structural probes to show that these solid solutions are not thermodynamically stable, and their synthesis under thermodynamic control leads to a segregated mixture of yellow one-dimensional DMAPbI_3_ phase and δ-CsPbI_3_. We show that mixed-cation DMA_*x*_Cs_1–*x*_PbI_3_ perovskite phases only form when they are kinetically trapped by rapid antisolvent-induced crystallization. We explore the energetics of DMA incorporation into CsPbI_3_ using first-principles calculations and molecular dynamics simulations and find that this process is energetically unfavorable. Our results provide a complete atomic-level picture of the mechanism of DMA-induced stabilization of the black perovskite phase of CsPbI_3_ and shed new light on this deceptively simple material.

Organic–inorganic halide perovskite solar cells (PSCs) have been developed within a decade to achieve remarkable power conversion efficiencies (PCEs) of over 25%.^1,2^ However, their thermal stability has been a major bottleneck due to the volatility of the organic components at elevated temperatures, which leads to irreversible degradation of device performance over time.^3−7^ All-inorganic PSCs based on cesium lead halides have attracted significant attention owing to their higher thermal stability and have reached PCEs on the order of 20%, albeit typically with various organic additives.^8−16^ CsPbI_3_ has been identified as one of the most promising solar cell materials due to its bandgap (*E*_g_ = 1.71 eV) which is close to the radiative efficiency limit.^8,17^ However, its perovskite α phase (*Pm*3̅*m*) is thermodynamically stable only above ca. 300 °C. On cooling, it transforms first to the tetragonal β phase (*P*4/*mbm*) and then the orthorhombic γ phase (*Pbnm*) with distorted corner-sharing octahedra (Figure 1A).^8,18,19^ The γ phase is metastable at room temperature and readily transforms to an orthorhombic nonperovskite δ-phase (*Pnma*). The metastable perovskite phase (γ-CsPbI_3_) has been stabilized using several strategies to date to enable its use in optoelectronic devices. These strategies include incorporating Br^–^ and F^–^;^13,16,20−22^ solvent-controlled growth;^23,24^ the use of intermediate phases;^25^ doping with metal ions such as Bi^3+^,^26,27^ Sn^2+^,^21^ Sb^2+^,^28^ and Eu^2+^;^16,29^ passivation with small organic molecules;^30−32^ and addition of hydroiodic acid (HI) to the precursor solution.^33−35^ The last strategy has been remarkably successful and has been adopted as the primary method to fabricate all-inorganic PSCs based on CsPbI_3_.^36−38^ In 2018, Ke et al. reported that the addition of HI catalyzes the acidic hydrolysis of the commonly used solvent, dimethylformamide (DMF), leading to dimethylammonium (DMA) as a degradation product. DMA was thought to incorporate into the perovskite structure as an A-site cation leading to DMA_*x*_Cs_1–*x*_PbI_3_ phases.^34^ Further, Wang et al. showed that DMA can affect CsPbI_3_ crystallization kinetics and thin film morphology.^38^ DMA has also been used in hybrid PSCs in an attempt to modulate electronic properties,^39^ stability,^36−38,40^ and efficiency.^34,36−38^ However, the speciation and microscopic mechanism of action of DMA in mixtures with CsPbI_3_ in the solid state have been elusive. More recently, Marshall et al. concluded that mixed-cation DMA_*x*_Cs_1–*x*_PbI_3_ phases form using a combination of optical spectroscopies and XRD.^41^ Recent progress in characterizing the role of DMA in perovskite solar cells has recently been reviewed.^62^ While those techniques provide insight into the long-range structure and bulk properties of materials, there is a critical need to investigate the local structure of the individual components to assess their structure and role at the atomic level.

**Figure 1:**
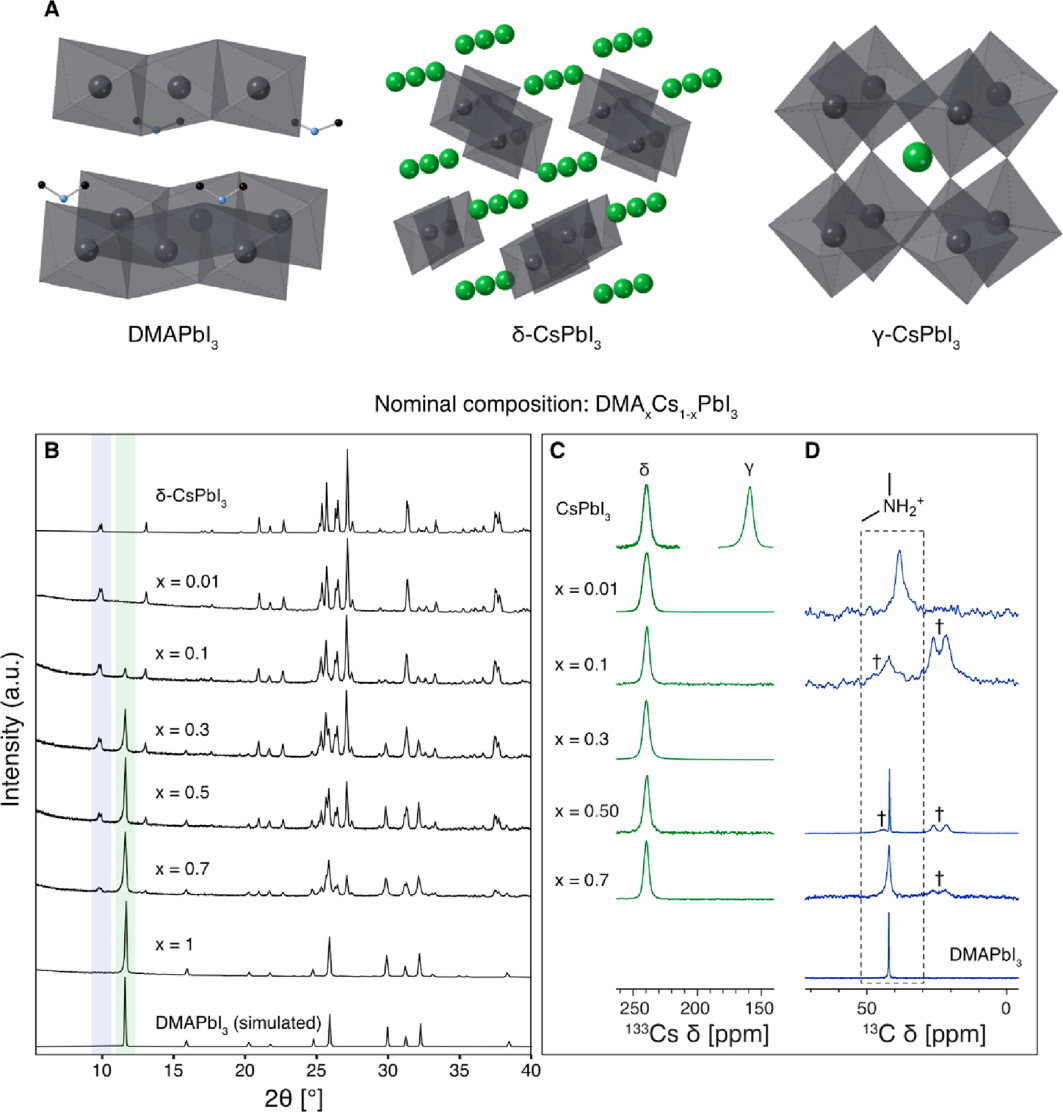
Characterization of the DMA_*x*_Cs_1–*x*_PbI_3_ materials. (A) Crystal structures of the compositional end members showing the PbI_6_ connected octahedra in gray, Cs^+^ in green, and DMA cations (C, black; N, blue); (B) powder XRD (see Figure S2 for enlarged view), (C) ^133^Cs MAS, and (D) ^1^H–^13^C CPMAS solid-state NMR spectra. All experiments were carried out at room temperature (ca. 294 K). The NMR spectrum of the *x* = 0.01 material was recorded using a Hahn echo to avoid baseline distortions. † indicates trace (<2 wt % based on the ^1^H spectrum) polypropylene (PP) used as the grinding jar material, peaks at 21.5, 26.1, and 44.3 ppm. The *x* = 0.01 material was ground in an agate jar to avoid PP contamination. The full ^133^Cs spectra and further experimental details are given in Figure S1 and Tables S1 and S3.

Solid-state NMR spectroscopy has emerged as a powerful local structure characterization technique to determine the speciation of dopants.^42−44^ Here, we use it to show that the formation of mixed-cation iodoplumbates of DMA and Cs, i.e., compositions with the nominal formula DMA_*x*_Cs_1–*x*_PbI_3_, is determined by an interplay of kinetic and thermodynamic reaction control factors. We experimentally show that thermodynamic reaction conditions (mechanosynthesis, solution processing without an antisolvent) lead to materials completely segregated into δ-CsPbI_3_ and DMAPbI_3_, while kinetic reaction control (spin coating with rapid crystallization induced by antisolvent dripping) leads to a mixed-cation solid solution. From this, we conclude that the atomic-level mechanism of γ-CsPbI_3_ stabilization with DMAI in thin films processed with an antisolvent involves incorporation of DMA into the perovskite structure. We elucidate and rationalize the composition of the resulting materials using a combination of long-range (X-ray diffraction) and local structure (solid-state NMR) probes and density functional theory (DFT)-based calculations and molecular dynamics (MD) simulations.

We first focus our attention on compositions corresponding to the nominal formula DMA_*x*_Cs_1–*x*_PbI_3_ (*x* = 0.01, 0.10, 0.30, 0.50, 0.70, 1.0) prepared using solid-state mechanosynthesis followed by annealing at 100 °C (see Methods in the SI). All of the resulting polycrystalline powders were yellow. Powder X-ray diffraction (pXRD) data show that the mixed DMA/Cs samples are mixtures of two nonperovskite hexagonal phases: DMAPbI_3_ and δ-CsPbI_3_ (Figure 1B). The composition-dependent evolution can be readily followed in the 2θ = 9–12° region where δ-CsPbI_3_ yields reflections at 9.8° and 10.0°, while DMAPbI_3_ has a reflection at 11.6°. The diffractograms of mixed DMA/Cs compositions correspond to mixtures of δ-CsPbI_3_ and DMAPbI_3_, with no new mixed-cation phases being present.

To gain a more detailed picture of Cs/DMA mixing, we elucidate the local structure of Cs and DMA using magic-angle-spinning (MAS) ^133^Cs and ^13^C solid-state NMR, respectively. ^133^Cs NMR is particularly useful for studying Cs-containing metal halide perovskites (MHPs) as it can be used to evidence Cs incorporation into hybrid MHPs^46^ since its shift strongly depends on the structure topology and halide composition.^45^Figure 1C shows the ^133^Cs MAS NMR spectra of the materials. The reference CsPbI_3_ sample was annealed at 300 °C before the measurement to capture the metastable γ perovskite phase (158 ppm). The transformation back to the orthorhombic δ phase (240 ppm) occurs on the time scale of minutes (half-life of γ-CsPbI_3_ at RT is about 30 min),^45^ so it possible to record a high-quality spectrum of each phase by taking a measurement immediately after annealing (phase-pure γ-CsPbI_3_) and after a few hours (phase-pure δ-CsPbI_3_). For comparison, a spectrum of this material midtransition is shown as a reference in Figure 2A. ^133^Cs spectra of the compositions formally denoted as DMA_*x*_Cs_1–*x*_PbI_3_ (*x* = 0.01, 0.1, 0.3, 0.5, 0.7) show a single peak corresponding to δ-CsPbI_3_ with no substantial shift or line width variation as a function of the DMA/Cs ratio (see also Table S1). The γ phase was not detected in any of the samples, consistent with their yellow appearance. To elucidate the speciation of DMA, we recorded room-temperature ^13^C cross-polarization (CP) MAS NMR spectra (Figure 1D). While the ^13^C chemical shift of DMA in the DMA_*x*_Cs_1–*x*_PbI_3_ (*x* = 0.1, 0.3, 0.5, 0.7) compositions is consistent with it being present as DMAPbI_3_, in agreement with the XRD data, we found remarkable variation in line width, which is uncorrelated with the DMA/Cs ratio (Table S2). For example, the line widths are 0.224 ± 0.006 ppm for DMAPbI_3_, 1.35 ± 0.03 ppm for DMA_0.7_Cs_0.3_PbI_3_, and 3.48 ± 0.04 ppm for DMA_0.01_Cs_0.99_PbI_3_. Since broader line widths correspond to more disordered local environments, we attribute this effect to the presence of nanosized regions and disorder of DMAPbI_3_ formed as a result of mechanosynthesis. Interestingly, in the case of the *x* = 0.01 material, the ^13^C signal of DMA is shifted to lower frequencies, indicating a substantially different local environment. This effect may result from the interaction of the nanosized grains of DMAPbI_3_ with the surface of δ-CsPbI_3_ owing to the high level of dispersion of the hybrid phase within the all-inorganic matrix. The effect is only visible in the most dilute material where we recorded the ^13^C spectrum over 214 h. We suggest that this effect could be further investigated using surface-enhanced NMR spectroscopy.^51^ Taken together, these XRD and solid-state NMR results show that DMA has no propensity to form thermodynamically stable mixed-cation phases with CsPbI_3_ and instead forms DMAPbI_3_, i.e., the two cations do not mix at the atomic level. However, the formation of a black perovskite phase in this phase diagram has been repeatedly reported by multiple groups using solution synthesis,^33,34^ which led us to investigate solution-processed DMA_0.20_Cs_0.80_PbI_3_. We chose this composition because it has a high DMA/Cs ratio while still falling within the stability range of the solid solution reported by Marshall et al.^41^ While we have previously shown that qualitative chemical reactivity tends to be identical in solution and mechanosynthesized materials,^46,47^ it appears that in this case solution processing is key to the formation of the black perovskite phase. We first attempted to make the solution-processed material by drop casting without the use of an antisolvent, and it behaved in analogy to the materials made by mechanosynthesis; i.e., we observed complete phase segregation into δ-CsPbI_3_ and DMAPbI_3_ (Figure 2A,C). However, all previous works reporting DMA-assisted formation of a stable black phase used an antisolvent in their deposition process. The role of an antisolvent is to induce rapid crystallization of an intermediate phase by reducing its solubility, which is transformed into the perovskite phase during annealing. Those results suggested to us that the use of an antisolvent may be key to understanding the CsI-DMAI-PbI_2_ phase diagram. After optimizing the deposition process (see the Experimental Section in the SI for details), we found that chlorobenzene reproducibly yields black films, which are stable, if humidity is strictly excluded (Figure 2B). The ^133^Cs MAS NMR spectrum of this black form shows a new very broad peak centered at about 100 ppm with a line width of 42 ppm (Figure 2A). This NMR signature is reminiscent of Cs^+^ incorporated into FAPbI_3_ (Figure 2A, bottom), with the remarkably large line width resulting from substantial static disorder, i.e., the presence of a distribution of different nearest and next-nearest neighbor local environments. For example, in the cubic perovskite aristotype CsPbI_3_, Cs has six nearest neighbors at 6.3 Å (neighboring cubooctahedra), 12 nearest neighbors at 8.9 Å (across the [PbI_6_]^4–^ vertices), and eight more at 10.9 Å (across the [PbI_6_]^4–^ octahedra). Including all the next-nearest neighbors within the 20 Å radius, it has a total of 146 Cs^+^ ions surrounding it, each of which can be replaced by DMA, leading to a slight change in ^133^Cs shift. The convolution of these possibilities leads to the experimentally observed broad line shape. Despite all of the precautions taken (handling inside an argon glovebox, transfer of the rotor in an airtight Schlenk flask, spinning using dry nitrogen), we found that this phase disappears within 3–4 h of starting the measurement. Figure S3 shows the evolution of this spectrum as a function of time. We were unable to record a ^13^C spectrum of this material (no signal after 8 h of acquisition), presumably because of the small amount of material and substantial disorder of the degraded phase leading to signal broadening. The spectrum shown in Figure 2A is a sum of spectra taken during the first 8 h of the measurement. Another interesting aspect of this spectrum is that the signal corresponding to δ-CsPbI_3_ has a broad underlying component, which corresponds to a δ-CsPbI_3_ in which some Cs was replaced by DMA. This result is similar to what we have previously observed in Cs-rich FAPbI_3_ compositions such as FA_0.16_Cs_0.84_PbI_3_.^48^ These results evidence that DMA can be incorporated into the perovskite phase of CsPbI_3_, with the prerequisite being fast antisolvent-induced crystallization, which leads to kinetic stabilization of the metastable phase. On the other hand, when the material is prepared under thermodynamic conditions (mechanosynthesis, slow solvent evaporation), complete phase segregation into δ-CsPbI_3_ and DMAPbI_3_ results. This behavior contrasts with that observed for Cs/FA and Cs/GUA, which yield mixed-cation iodoplumbate phases under thermodynamic conditions.^46,48,49,61^ The importance of kinetic reaction control also has potential bearing on the role and speciation of Rb^+^ and K^+^ in hybrid MHPs doped with these cations, where we have previously observed complete segregation of the inorganic dopants under thermodynamic control.^46,50^ It is also noteworthy that the overall structure topology affects the incorporation of small organic cations into halide perovskite cages. For example, while large cations, such as DMA and GUA do not form 3D perovskites on their own owing to their large size, they can be incorporated into the cubooctahedral space in 2*D*/3D Ruddlesden–Popper phases.^63−65^ The lack of thermodynamic stability of Cs/DMA iodoplumbate phases can be rationalized by the difference in ionic radii of Cs and DMA. The smaller Cs is replaced by the larger DMA, imposing distortions in the lattice. On increasing the DMA/Cs ratio, the volume of the unit cell is expected to increase proportionally. On the one hand, the incorporation of DMA alleviates the initial strain in γ-CsPbI_3_. On the other, because of the antibonding character of the valence band maximum, the reduction of the lattice distortion leads to an increase in the overlap of the I p and Pb s orbitals, which in turn leads to destabilization of the mixed-cation perovskite phase. We have previously also observed this phenomenon in Cs-rich Cs_*x*_FA_1–*x*_PbI_3_ solid solutions.^48^ For reference, on the basis of Bader volume calculations, DMA has an effective radius of 2.67 A, while MA and FA have effective radii of 2.37 and 2.48 Å, respectively. Guanidinium (GUA), which has been shown to form mixed GUA/MA and GUA/FA 3D perovskite phases, has an effective radius of 2.68 Å.^61^ Finally, we also noticed that the ^133^Cs shift of the Cs^+^ inside the perovskite phase is correlated with the content of the organic cation present in the solid solution (Figure 2D). There are two regions corresponding to stable A_*x*_Cs_1–*x*_PbI_3_ solid solutions: 0–25 mol % (A = DMA, kinetic stability) and >70 mol % (A = FA, thermodynamic stability). Interestingly, there are currently no known organic cations that lead to stable A_*x*_Cs_1–*x*_PbI_3_ phases for *x* in the 25–70 mol % range.

**Figure 2:**
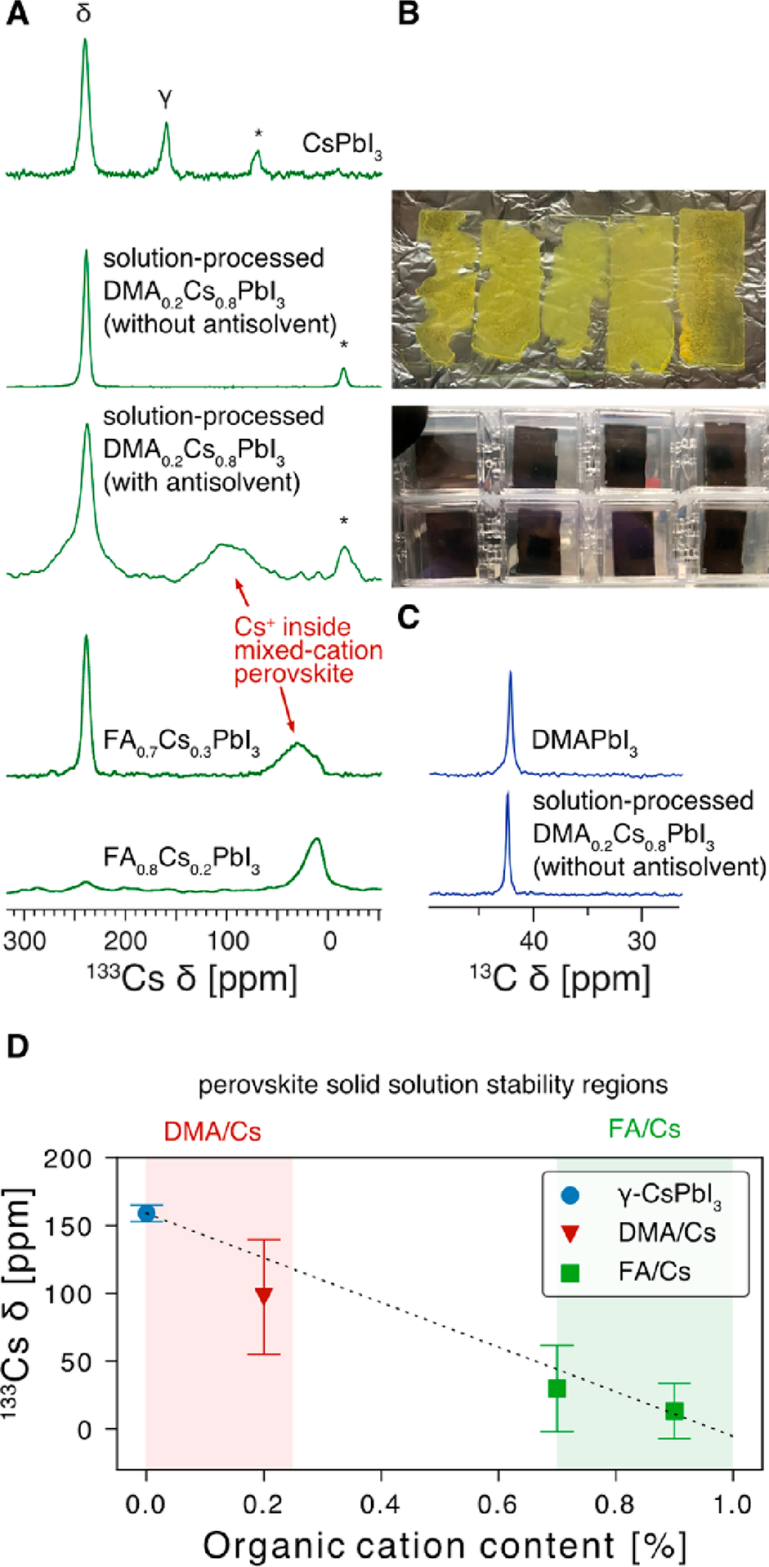
Characterization of DMA_0.2_Cs_0.8_PbI_3_ materials prepared by solution processing. (A) ^133^Cs MAS solid-state NMR spectra of DMA_0.2_Cs_0.8_PbI_3_ made with and without the use of an antisolvent (chlorobenzene). Rapid antisolvent-induced crystallization leads to kinetic trapping of a mixed-cation perovskite with DMA incorporated into the perovskite structure. Its NMR signature its similar to that previously observed for FA_*x*_Cs_1–*x*_PbI_3_ solid solutions (Adapted with permission from *J. Am. Chem. Soc.***2017**, *139* (40), 14173–14180. Copyright 2017, American Chemical Society). Asterisks indicate spinning sidebands. (B) Photographs of films made with and without the use of an antisolvent. (C) ^1^H–^13^C CPMAS solid-state NMR spectrum of DMA_0.2_Cs_0.8_PbI_3_ showing that DMA is only present as DMAPbI_3_. All experiments were carried out at room temperature (ca. 294 K). (D) Correlation between the ^133^Cs shift of black perovskite solid solutions of A_*x*_Cs_1–*x*_PbI_3_ where A = DMA and FA. The error bars are taken as the corresponding full width at half-maximum (fwhm) values. The solid-solution stability regions are indicated based on refs (41) and (46). The linear weighed regression equation is δ_Cs_ [ppm] = −164*x* + 159. Further experimental details are given in Figure S1 and Tables S2 and S3.

To gain further insight into the potential miscibility of DMA and Cs iodoplumbates, we next studied their mixing free energy using DFT calculations.^52,53^ We used the Perdew–Burke–Ernzerhof (PBE) functional^54^ to determine the relative stability of perovskite and nonperovskite phases of hypothetical DMA_*x*_Cs_1–*x*_PbI_3_ mixtures at 0 K with up to 50 mol % of Cs replaced by DMA according to
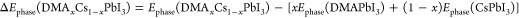


We found that the energy difference between various substitution patterns at any concentration is only on the order of 0.01 eV per stoichiometric unit for both the γ and δ phases. This small amount justifies the use of the analytical formula for ideal alloys to estimate the mixing entropy contribution to the free energy.^55^
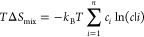
where *k*_B_ is the Boltzmann constant, *T* is the temperature, *n* is the number of components, and *c*_*i*_ is the atomic fraction of component *i*. In the case of DMA_*x*_Cs_1–*x*_PbI_3_ mixtures, the above equation leads to the following:
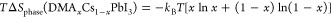
where *x* is the mol % of DMA incorporated into the CsPbI_3_ structure.

Figure 3A shows the energetic and entropic contributions to the free energy of mixing as a function of *x* for the two phases. For the γ phase and low DMA molar fractions, the replacement of Cs with DMA induces a negligible or slightly positive energetic contribution on the order of 0.01 eV per stoichiometric unit, i.e., energies on the same order of magnitude as the variations between different substitution patterns. Since the values of the mixing free energy for molar fractions of DMA below 37.5 mol % lie within the intrinsic error of the calculations, it is not possible to unambiguously conclude if DMA/Cs mixing in the γ phase leads to marginal stabilization relative to the single-cation end members. On the other hand, for DMA fractions larger than 37.5 mol %, both energetic and entropic contributions favor the formation of mixed-cation phases. In contrast, in the δ phase, cation mixing leads to significant energetic destabilization for all DMA_*x*_Cs_1–*x*_PbI_3_ compositions. The mixing entropy contribution to the free energy calculated at room temperature (298 K) is not sufficient to compensate for this penalty; i.e., in the δ phase, all mixtures in this molar fraction range are predicted to be unstable with respect to demixing into single-cation phases. For a possible reversion of relative phase stability between the γ and δ-phases in the mixed compounds, the energetic stabilization of the γ phase and the simultaneous destabilization of the δ phase would have to be large enough to compensate for the initial energy penalty between the two phases, which however turns out not to be the case (Figure 3B).

**Figure 3:**
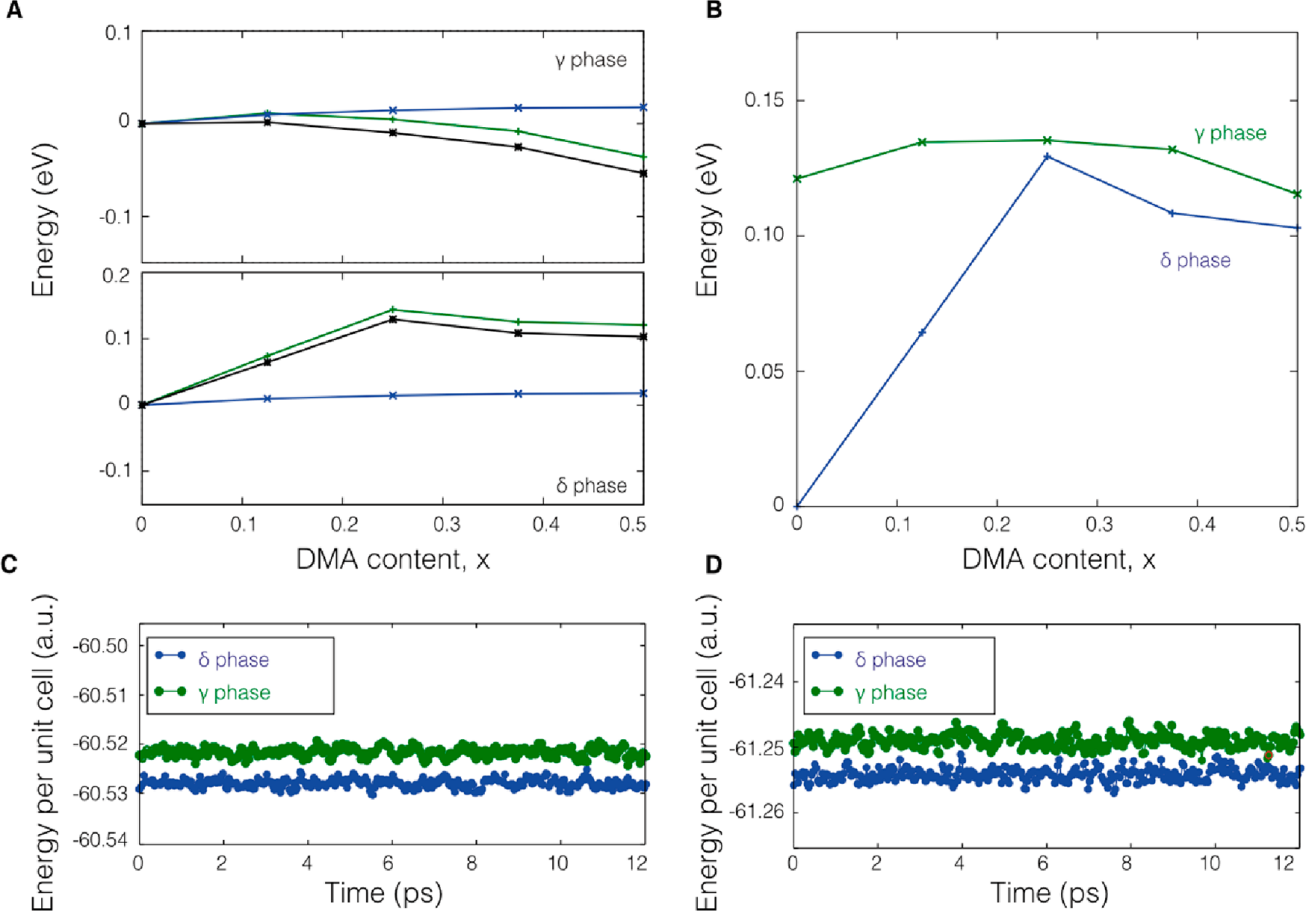
Energetics of Cs/DMA mixing in the γ and δ phases. (A) Variation of the free energy of mixing expressed as Δ*F* = Δ*E* – *T*Δ*S* (black line), the internal energy, Δ*E* (green line), and the mixing entropy contribution, *T*Δ*S* (for *T* = 298 K, blue line), as a function of the molar fraction of DMA in DMA_*x*_Cs_1–*x*_PbI_3_ mixtures for the perovskite and δ phases, respectively. (B) Variation of the free energy of mixing plotted relative to the pure δ phase as a reference. Energies are given per stoichiometric unit. Potential energy per stoichiometric unit for perovskite and δ phases from first-principles MD simulations in a constant-temperature, constant-volume (NVT) ensemble at 300 K for (C) Cs_0.625_DMA_0.375_PbI_3_ and (D) Cs_0.5_DMA_0.5_PbI_3_. The *y* axis shows energy per stoichiometric unit. Full details of the calculation methods are given in the SI.

Table 1 shows the relative energetics per stoichiometric unit between the δ and the γ phases at 0 K. The δ phase remains energetically preferred at 0 K, but the energy difference is substantially reduced upon mixing, consistent with the energetic trends in the mixing free energies.

**Table 1:** Relative Energetics Given by Δ*E* = *E*_γ_ – *E*_δ_ per s.u. of the γ Relative to the δ Phase in DMA_*x*_Cs_1–*x*_PbI_3_

*x* (mol %)	Δ*E* (eV)
0	0.12
12.5	0.07
25	0.01
37.5	0.02
50	0.01

The stability of mixed component phases at room temperature can be substantially affected by vibrational entropy which is absent in the calculations carried out at 0 K. In the case of lead halide perovskites, the calculation of vibrational corrections is not trivial and leads to the appearance of imaginary modes in harmonic and quasi-harmonic phonon calculations.^56^ This artifact has been addressed by including anharmonic phonon–phonon interactions in halide double perovskites.^57,58^ However, in all-inorganic lead halide perovskites, vibrational instabilities are present that are associated with octahedral tilting in the high-temperature phase.^59,60^ Consequently, we approached the question of assessing relative phase stabilities at finite temperatures by carrying out direct MD simulations (full details are given in SI). Figure 3C and D show potential energies per stoichiometric unit for γ and δ phases of Cs_0.625_DMA_0.375_PbI_3_ and Cs_0.5_DMA_0.5_PbI_3_ from first-principles MD simulations at 300 K. Figure 3C and D show that the δ phase remains thermodynamically more stable than the perovskite phase even when finite temperature effects are fully taken into account. Given the time scale of first-principles MD simulations (of a few picoseconds) and considering that the systems do not contain any vacancies that might accelerate demixing, no spontaneous phase segregation toward the pure phases is expected to be observed, although the calculated mixing free energies show that all mixtures in the δ-phase are unstable with respect to demixing.

In conclusion, we have assessed the possibility of incorporating DMA into the CsPbI_3_ lattice using a combination of long-range and local structure probes and static and dynamic first-principles calculations. Our results show a remarkable dependence on the reaction conditions, with kinetic control leading to stabilization of the mixed-cation DMA_*x*_Cs_1–*x*_PbI_3_ perovskite phase and thermodynamic control resulting in complete phase segregation into a physical mixture of δ-CsPbI_3_ and DMAPbI_3_. These experimental findings are rationalized by DFT calculations, which show that, although cation mixing in the perovskite phase is possible, the overall, thermodynamically most stable phase is the demixed δ-phase. We contend that further multimodal studies into the chemical transformations of CsPbI_3_ and its solid solutions are urgently needed to improve our understanding of these materials.

## Supporting Information Available

Materials and methods and details of the solid-state NMR measurements (PDF)

## Notes

All data presented here (raw NMR and XRD data, and DFT and MD input files) can be accessed at the following DOI: 10.5281/zenodo.6903645 and it is available under the license CC-BY-4.0 (Creative Commons Attribution-ShareAlike 4.0 International).
