# Supplementary material for: Interplay of Kinetic and Thermodynamic Reaction Control Explains Incorporation of Dimethylammonium Iodide into CsPbI3
Source: ACS Energy Lett. 2022 Jul 26;7(8):2745–52. doi: 10.1021/acsenergylett.2c00877 (PMC9380017; doi:10.1021/acsenergylett.2c00877)
Supplement: Supplementary file 1 — nz2c00877_si_001.pdf [file nz2c00877_si_001.pdf]

Supporting information

Interplay of kinetic and thermodynamic reaction control explains reactivity of dimethylammonium iodide with $CsPbI_3$

Aditya Mishra,[1] *Dominik J. Kubicki,*[1,2]* *Ariadni Boziki,*[3] Rohit D. Chavan,[4] *Mathias Dankl,*[3] *Marko Mladenović,*[3] Daniel Prochowicz,[4] *Clare P. Grey,*[4] *Ursula Rothlisberger,*[3]*, *Lyndon Emsley*[1]*

[1]Laboratory of Magnetic Resonance, Institut des Sciences et Ingénierie Chimiques, École Polytechnique Fédérale de Lausanne (EPFL), 1015 Lausanne, Switzerland
[2]Department of Physics, University of Warwick, CV4 7AL, Coventry, UK
[3]Laboratory of Computational Chemistry and Biochemistry, Institut des Sciences et Ingénierie Chimiques, École Polytechnique Fédérale de Lausanne (EPFL), 1015 Lausanne, Switzerland
[4]Institute of Physical Chemistry, Polish Academy of Sciences, Kasprzaka 44/52, 01-224 Warsaw, Poland
[5]Department of Chemistry, University of Cambridge, Lensfield Road, Cambridge, CB2 1EW, UK

Email Correspondence: dominik.kubicki@warwick.ac.uk, ursula.roethlisberger@epfl.ch, lyndon.emsley@epfl.ch

Experimental 2
Supplementary Figures 5
NMR acquisition and processing details 8
References 9

Experimental

Materials. The following materials were used: dimethylammonium iodide (TCI, >98%), CsI (Sigma, 99.9%), PbI_2 (Sigma, 99%), chlorobenzene (Sigma, anhydrous 99.8%), diethyl ether (Sigma, 99.9%), anhydrous N,N-dimethylformamide (Sigma, 99.8%), dimethyl sulfoxide (Sigma, 99.9%).

Perovskite mechanosynthesis. The materials were prepared using mechanosynthesis following the previously published protocol,[1–6] which was as follows. The precursors were stored and weighed out (±1 mg) into either an agate grinding jar (10 mL) containing an agate ball (⌀10 mm) or 2 mL polypropylene (PP) Eppendorf vial containing a stainless steel (⌀4 mm) ball under argon. We found that both types of grinding vessels lead to the same products, but the latter introduces trace amounts of PP through abrasion, which lead to background in the ^{13}C CP spectra. The following stoichiometries were used:

$CsPbI_3$: CsI (129.9 mg, 0.50 mmol), and PbI_2 (230.5 mg, 0.50 mmol),
$DMA_{0.01}Cs_{0.99}PbI_3$: DMAI (0.9 mg, 0.005 mmol), CsI (128.6 mg, 0.495 mmol), and PbI_2 (230.5 mg, 0.50 mmol),
$DMA_{0.10}Cs_{0.90}PbI_3$: DMAI (8.7 mg, 0.05 mmol), CsI (116.9 mg, 0.45 mmol), and PbI_2 (230.5 mg, 0.50 mmol),
$DMA_{0.30}Cs_{0.70}PbI_3$: DMAI (26.0 mg, 0.15 mmol), CsI (90.9 mg, 0.35 mmol), and PbI_2 (230.5 mg, 0.50 mmol),
$DMA_{0.50}Cs_{0.70}PbI_3$: DMAI (43.4 mg, 0.25 mmol), CsI (65.0 mg, 0.25 mmol), and PbI_2 (230.5 mg, 0.50 mmol),
$DMA_{0.70}Cs_{0.70}PbI_3$: DMAI (60.6 mg, 0.35 mmol), CsI (39.0 mg, 0.15 mmol), and PbI_2 (230.5 mg, 0.50 mmol),
$DMAPbI_3$: DMAI (86.5 mg, 0.5 mmol), and PbI_2 (230.5 mg, 0.50 mmol).

The combined precursors were ground in an electric ball mill (Retsch MM−400) for 30 minutes at a vibration frequency of 25 Hz. The resulting powders were scraped off the walls of the grinding jars, transferred into glass vials and annealed for 5 minutes at 100 °C to remove grinding-induced defects. In the case of $CsPbI_3$, the annealing temperature was 300 °C to induce the phase transition to the metastable perovskite phase, the sample was heated directly in a rotor using a hot-air blower outside the magnet and quickly transferred to the NMR probe for measurements. The annealing temperature was monitored by attaching the rotor to a thermocouple. Karmakar et al. have previously found that the half-life of the γ phase prepared in this way is on the order of 30 minutes (see Figure S8 therein), and our experience corroborates this result.

Precursor solution preparation. $Cs_{0.8}DMA_{0.2}PbI_3$ perovskite solution was prepared by dissolving CsI (208 mg), DMAI (34.6 mg) and PbI_2 (461 mg) in 1 mL of DMF:DMSO solution (4:1, v/v). The solution was stirred in a nitrogen glovebox at room temperature for 1 hour.

Deposition of solution processed $DMA_{0.2}Cs_{0.8}PbI_3$ (drop cast, without antisolvent). 2 mL of the precursor solution were drop cast using a pipette onto microscope slides kept at 100 °C on a hot plate inside an argon glovebox. The solution was allowed to evaporate over 20 minutes and the dry yellow powder was scraped off and packed into a rotor.

Deposition of solution processed $DMA_{0.2}Cs_{0.8}PbI_3$ (spin-coated, with antisolvent). Pre-cleaned FTO glasses were treated with UV-ozone for 15 min. and annealed at 150 °C for 60 min. The deposition process and subsequent handling were carried out inside an argon glove box. The perovskite solution was spin-coated onto the substrate at 1000 rpm for 10 s and continuously at 3000 rpm for 30 s. The antisolvent (CB, 200 μl) was dropped on the spinning substrate during the second spin-coating step at 15 s before the end of the procedure, followed by annealing at 100 °C for 5 min. The resulting black material was scraped off of the substrates (25 substrates in total) using a razor blade and transferred into a rotor. The sealed rotor was transported from the glovebox to the spectrometer inside an air-tight Schlenk flask and only exposed to ambient air for <1 minute, the time needed to load it into the probe where it was in dry nitrogen atmosphere. Despite these precautions, we found that the black perovskite phase transitioned to the non-perovskite yellow polymorph within 3-4 hours after starting the NMR measurement (Figure S3).

Antisolvent optimization. To fabricate stable high-quality black perovskite films, we tested two deposition approaches, with and without an antisolvent. We found that films fabricated without the use of an antisolvent do not form the black phase. We tested two antisolvents, chlorobenzene (CB) and diethyl ether (DEE). In our hands, the use of CB led to more stable and high-quality black films of $DMA_{0.2}Cs_{0.8}PbI_3$ compared to DEE.

Optimization of annealing temperature. We tested 3 approaches, all carried out inside a nitrogen glovebox:

1. Two-step annealing – the films were annealed for 2 min. at 60 °C and then 5 min at 100 °C on a hot plate. In this method, the films were not sufficiently stable (black phase retained about 1 hour)
2. High-temperature annealing - the films were annealed for 2 min at 100 °C and then 5 min at 180 °C on a hot plate. After the first step, the films were stable but after applying high temperature (180 °C), rapid films degradation started due to decomposition of the organic component (DMA^+). This result suggests that the high temperature (180 °C) is not suitable for the $DMA_{0.2}Cs_{0.8}PbI_3$ composition, in agreement with previous reports.
3. One-step annealing – the films were annealed for 5 min at 100 °C on a hot plate. In this method, the black phase was stable for at least 24 hours. This protocol was used to fabricate the films used in the solid-state NMR experiment. 25 films were fabricated and used in total.

Powder X-ray diffraction. Powder X-ray diffraction patterns were recorded on an X'Pert MPD PRO (Panalytical) diffractometer equipped with a ceramic tube (Cu anode, $\lambda = 1.54060$ Å), a secondary graphite (002) monochromator and an RTMS X'Celerator (Panalytical) in an angle range of $2\theta = 5°$ to 40°, by step scanning with a step of 0.02 degree.

NMR measurements. Solid-state MAS NMR spectra of ^{133}Cs (52.6 MHz) were recorded on a Bruker Avance Neo 9.4 T spectrometer equipped with a 4 mm MAS probe using 62.5 kHz RF field amplitude or a Bruker Avance Neo 11.7 T equipped with a 3.2 mm MAS probe using 50 kHz RF field amplitude (see Table S1 for details) and referenced to solid CsI (271.05 ppm).[7] The recycle delays to obtain quantitative spectra were set based on the previously measured T_1 values[3] and are given in Table S1. Room-temperature ^{13}C MAS and ^{1}H-^{13}C (100.9 MHz) CP MAS spectra were recorded on a Bruker Avance Neo 9.4 T spectrometer equipped with a 4.0 mm CPMAS probe and referenced to solid adamantane (38.48 ppm for the CH_2 signal).[8] 80 kHz ^{1}H decoupling was used. The rotors were spun using dry nitrogen.

Calculation details. DFT calculations[9,10] have been performed using the Quantum ESPRESSO suite of codes[11,12]. The generalized gradient approximation (GGA) to DFT in the PBE[13] formulation has been used for geometry and cell relaxations. Ultrasoft pseudopotentials have been used to model the interactions between valence electrons, core electrons and nuclei[14].The Kohn-Sham orbitals have been expanded in a plane wave basis set with a kinetic energy cutoff of 45 Ry and a density cutoff of 270 Ry. The Brillouin zone has been sampled with a Monkhorst Pack k-points grid[15] that varied from 3x3x2 points for the γ-phase to 3x3x2 for the δ-phase of $CsPbI_3$, and from 3x2x3 points for the perovskite phase to 2x2x3 for the δ-phase of $DMAPbI_3$. These choices are based on convergence tests for the total energy, the band gap, the pressure, the stresses and the atomic forces. To compare the absolute energy of Kohn-Sham orbitals and total energies, we followed the protocol suggested by Meloni et al.[16] in which the energies were aligned with respect to the energy of the Pb 5d semi-core state. For the preparation of the mixtures, Cs^+ has been successively replaced by DMA^+. In particular, for each concentration when allowed by the composition for both the γ and the δ phases, we considered 7 different possible replacements for the substituted cations, starting from the energetically most favorable structure of the lower DMA^+ content. The maximum standard deviation of the energy per stoichiometric unit for different substitution pattern is of the order of 0.01 eV for the γ and 0.014 eV for the δ phase, respectively. We have also performed calculations with different DMA^+ orientations, where the maximum standard deviation of the difference in energy per stoichiometric unit was of the order of 0.02 eV. Finally, to compare the mixed perovskite compounds with the corresponding pure ones, we considered different 3D perovskite $DMAPbI_3$ phases that we constructed by substituting either MA^+ or Cs^+ by DMA^+ in the cubic, tetragonal and orthorhombic phases of $MAPbI_3$ and the orthorhombic phase of $CsPbI_3$, considering also different orientations of the cations. We concluded that the structure with the lowest energy and thus the most stable one at 0 K is arising from the orthorhombic $CsPbI_3$ phase. Car-Parrinello MD simulations for trajectories of ~14 ps in the NVT ensemble at 300 K were performed using the CPMD 4.1 code[17] for systems of 140 atoms ($DMA_{0.375}Cs_{0.625}PbI_3$) and 160 atoms ($DMA_{0.5}Cs_{0.5}PbI_3$), respectively. The wave-function cutoff was set to 90 Ry and Goedecker normconserving pseudopotentials were used[18-20]. A time

step of 3 a.u. was used with a fictitious mass parameter of 400 a.u. The temperature was controlled by applying one Nosé-Hoover thermostat per atomic species with a coupling frequency of 1500 cm^{-1} [21-23]. The analysis of the relative energetics has been performed for equilibrated trajectories of ~12 ps.

Supplementary Figures

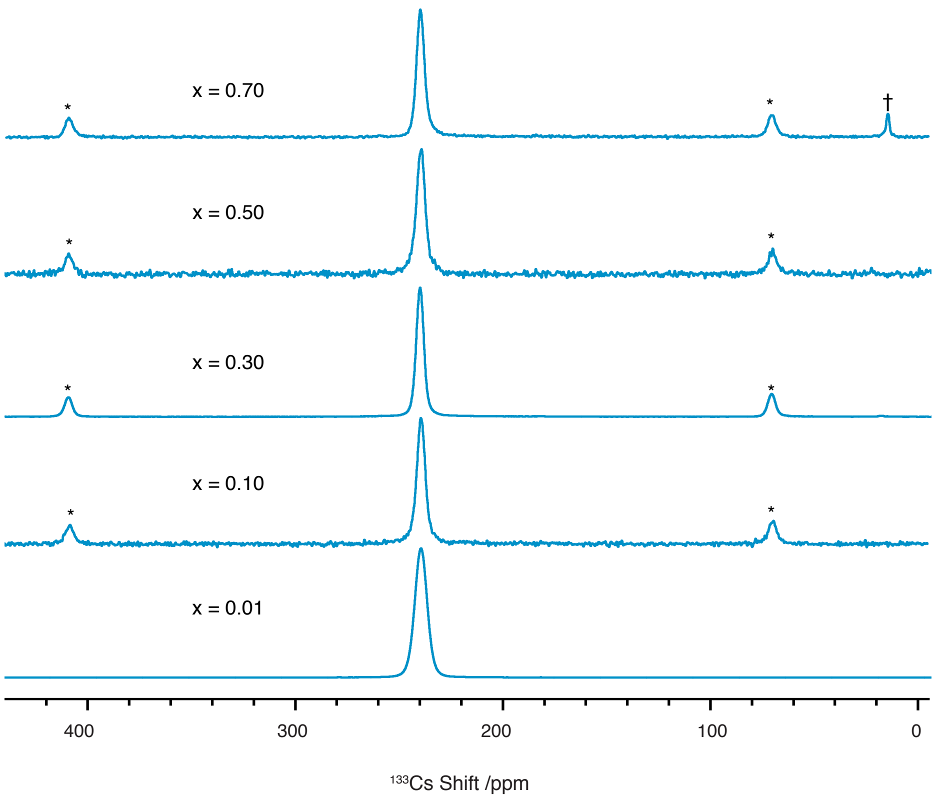

Figure S1. ^{133}Cs MAS NMR spectra of $DMA_xCs_{1-x}PbI_3$ materials. Asterisks (*) indicate spinning side-bands, the dagger (†) indicates an unidentified impurity.

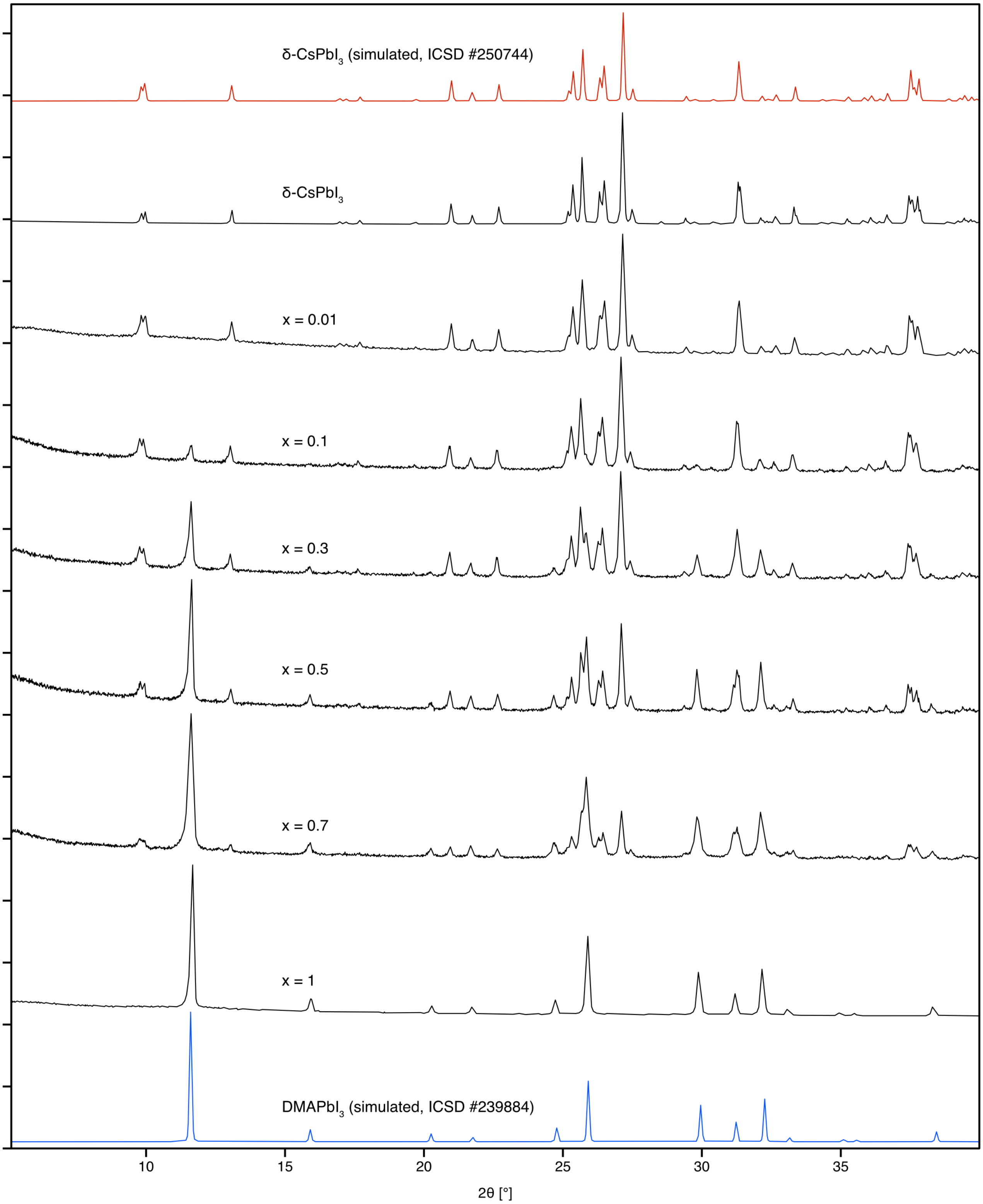

Figure S2. Enlarged view of the XRD data in Figure 1B, including a simulated pattern of δ-$CsPbI_3$.

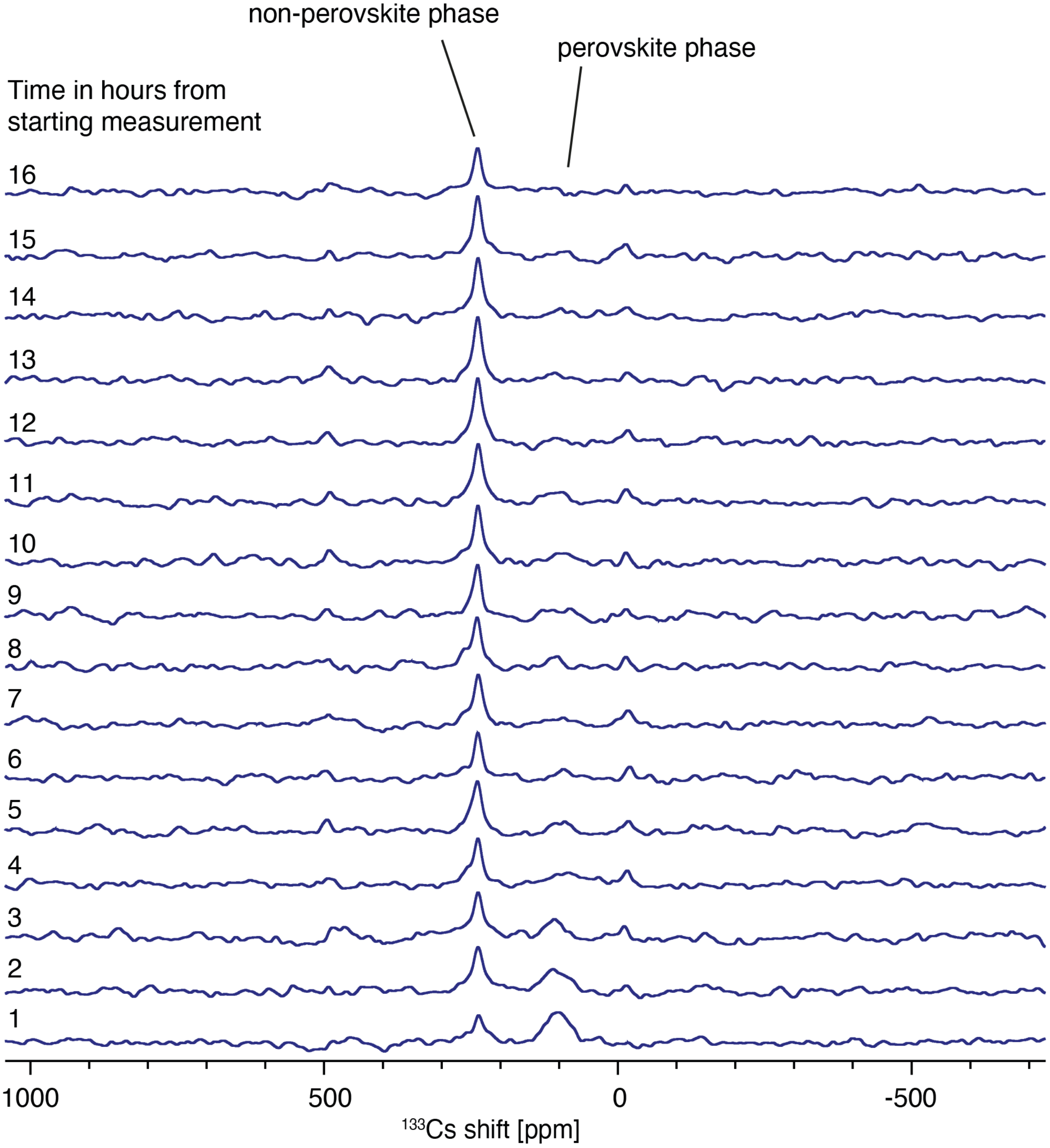

Figure S3. ^{133}Cs MAS NMR spectra of $DMA_{0.20}Cs_{0.80}PbI_3$ prepared as solution-processed thin films (25 substrates, spin-coated, with antisolvent) as a function of time. Each spectrum consists of 32 scans with a recycle delay of 110 s (total scan time 59 minutes). Further experimental details are given in Table S2.

NMR acquisition and processing details

Table S1. ^{133}Cs NMR acquisition and processing parameters for the $DMA_xCs_{1-x}PbI_3$ materials shown in Figure 1 of the main text. The FWHM values are given in ppm, in addition to Hz, to allow for a comparison between the two magnetic fields.

x	pulse sequence	Recycle delay [s]	# of scans	^{133}Cs shift [ppm]	^{133}Cs FWHM [Hz]	^{133}Cs FWHM [ppm]	Magnetic field [T]	MAS rate [kHz]	Apodization [Hz]
0 (δ)	Hahn echo	60	16	239.5	346 ± 2	5.27 ± 0.03	11.7	8	0
0 (γ)	Hahn echo	30	8	158.9	404 ± 4	6.15 ± 0.06	11.7	22	0
0.01	Bloch decay	500	12	239.4	301 ± 3	5.73 ± 0.05	9.4	14	0
0.10	Hahn echo	100	12	239.3	513 ± 5	4.35 ± 0.04	11.7	20	0
0.30	Hahn echo	100	452	239.7	435 ± 8	3.68 ± 0.06	11.7	20	0
0.50	Hahn echo	100	12	239.0	527 ± 6	4.46 ± 0.05	11.7	20	0
0.70	Hahn echo	100	1024	239.7	467 ± 7	3.95 ± 0.06	11.7	20	0

Table S2. ^{133}Cs NMR acquisition and processing parameters for the thin film $DMA_xCs_{1-x}PbI_3$ materials in Figure 2 of the main text. Line widths were measured without apodization.

x	pulse sequence	Recycle delay [s]	# of scans	^{133}Cs shift [ppm]	^{133}Cs FWHM [Hz]	^{133}Cs FWHM [ppm]	Magnetic field [T]	MAS rate [kHz]	Apodization [Hz]
0.20 (drop cast, without antisolvent)	Hahn echo	110	16	238.7	380 ± 5	4.84 ± 0.07	14.1	20	50
0.20 (spin coating, with antisolvent)	Hahn echo	32	256 (sum of 8 spectra)	238.6	520 ± 6 (δ phase); 3327 ± 54 (perovskite phase)	6.61 ± 0.07 (δ phase); 42.3 ± 0.7 (perovskite phase)	14.1	20	1000

Table S3. ^{13}C NMR acquisition and processing parameters for the $DMA_xCs_{1-x}PbI_3$ materials shown in Figures 1 and 2 of the main text. Line widths were measured without apodization.

x	pulse sequence	Recycle delay [s]	# of scans	^{13}C chemical shift [ppm]	^{13}C FWHM [Hz]	^{13}C FWHM [ppm]	Magnetic field [T]	MAS rate [kHz]	Apodization [Hz]
0.01	Hahn echo	5	153,856	38.1	351 ± 4	3.48 ± 0.04	9.4	12	50
0.10	CP	4	849	ca. 42	n/d	n/d	9.4	12	50
0.50	CP	7	46,882	41.8	24.7 ± 0.8	0.245 ± 0.008	9.4	12	0
0.70	CP	9	1,301	41.9	136 ± 3	1.35 ± 0.03	9.4	12	10
1.00	Bloch decay	5	128	42.1	22.6 ± 0.6	0.224 ± 0.006	9.4	12	0
0.20 (drop cast, without antisolvent)	CP	10	6,827	42.3	29 ± 1	0.188 ± 0.006	14.1	12	20

References

1. Prochowicz, D. *et al.* Mechanosynthesis of the hybrid perovskite $CH_3NH_3PbI_3$: Characterization and the corresponding solar cell efficiency. *J. Mater. Chem. A* **3**, 20772–20777 (2015).
2. Prochowicz, D. *et al.* Mechanosynthesis of pure phase mixed-cation $MA_xFA_{1-x}PbI_3$ hybrid perovskites: Photovoltaic performance and electrochemical properties. *Sustain. Energy Fuels* **1**, 689–693 (2017).
3. Kubicki, D. J. *et al.* Phase Segregation in Cs-, Rb- and K-Doped Mixed-Cation $(MA)_x(FA)_{1-x}PbI_3$ Hybrid Perovskites from Solid-State NMR. *J. Am. Chem. Soc.* **139**, 14173–14180 (2017).
4. Kubicki, D. J. *et al.* Cation Dynamics in Mixed-Cation $(MA)_x(FA)_{1-x}PbI_3$ Hybrid Perovskites from Solid-State NMR. *J. Am. Chem. Soc.* **139**, 10055–10061 (2017).
5. Prochowicz, D., Saski, M., Yadav, P., Grätzel, M. & Lewiński, J. Mechanoperovskites for Photovoltaic Applications: Preparation, Characterization, and Device Fabrication. *Acc. Chem. Res.* **52**, 3233–3243 (2019).
6. Kubicki, D. J. *et al.* Formation of Stable Mixed Guanidinium–Methylammonium Phases with Exceptionally Long Carrier Lifetimes for High-Efficiency Lead Iodide-Based Perovskite Photovoltaics. *J. Am. Chem. Soc.* **140**, 3345–3351 (2018).
7. Hayashi, S. & Hayamizu, Kikuko. Accurate determination of NMR chemical shifts in alkali halides and their correlation with structural factors. *Bull. Chem. Soc. Jpn.* **63**, 913–19 (1990).
8. Morcombe, C. R. & Zilm, K. W. Chemical shift referencing in MAS solid state NMR. *J. Magn. Reson.* **162**, 479–486 (2003).
9. Hohenberg, P.; Kohn, W. Inhomogeneous Electron Gas. *Phys. Rev.* **1964**, *136* (3B), B864–B871. https://doi.org/10.1103/PhysRev.136.B864.
10. Kohn, W.; Sham, L. J. Self-Consistent Equations Including Exchange and Correlation Effects. *Phys. Rev.* **1965**, *140* (4A), A1133-A1138. https://doi.org/10.1103/PhysRev.140.A1133.

11. Perdew, J. P.; Burke, K.; Ernzerhof, M. Generalized Gradient Approximation Made Simple. *Phys. Rev. Lett.* **1996**, *77* (18), 3865-3868. https://doi.org/10.1103/PhysRevLett.77.3865.
12. Giannozzi, P.; Baroni, S.; Bonini, N.; Calandra, M.; Car, R; Cavazzoni, C.; Ceresoli, D.; Chiarotti, G. L.; Cococcioni, M. et. al. QUANTUM ESPRESSO: a modular and open-source software project for quantum simulations of materials. *J. Phys.: Condens. Matter.* **2009**, *21* (39), 395502. https://doi.org/10.1088/0953-8984/21/39/395502
13. Giannozzi, P.; Andreussi, O.; Brumme, T.; Bunau, O.; Buongiorno, N. M.; Calandra, M.; Car, R.; Cavazzoni, C.; Ceresoli, D. et. al. Advanced capabilities for materials modelling with Quantum ESPRESSO. *J. Phys. Condens. Matter.* **2017**, *29* (46), 465901. https://doi.org/10.1088/1361-648X/aa8f79.
14. Dal Corso, A. Pseudopotentials periodic table: From H to Pu. *Comput. Mater. Sci.* **2014**, *95*, 337-350. https://doi.org/10.1016/j.commatsci.2014.07.043.
15. Monkhorst, H. J.; Pack, J. D.Special points for Brillouin-zone integrations. *Phys. Rev. B.* **1976**, *13* (12), 5188-5192. https://doi.org/10.1103/PhysRevB.13.5188.
16. Meloni, S.; Palermo, G.; Ashari-Astani, N.; Grätzel, M.; Rothlisberger, U. Valence and conduction band tuning in halide perovskites for solar cell applications. *J. Mater. Chem. A.* **2016**, *4* (41), 15997-16002. https://doi.org/10.1039/C6TA04949D.
17. CPMD, Copyright IBM Corp 1990-2015, Copyright MPI für Festkörperforschung Stuttgart 1997-2001. Online; accessed 1 July 2019, http://www.cpmd.org/, 1990.
18. Goedecker, S.; Teter, M.; Hutter, J. Separable dual-space Gaussian pseudopotentials. *Phys. Rev. B.* **1996**, *54* (3), 1703-1710. https://doi.org/10.1103/PhysRevB.54.1703.
19. Hartwigsen, C.; Goedecker, S.; Hutter, J. Relativistic separable dual-space Gaussian pseudopotentials from H to Rn. *Phys. Rev. B.* **1998**, *58* (7), 3641-3662. https://doi.org/10.1103/PhysRevB.58.3641.
20. Krack, M. Pseudopotentials for H to Kr optimized for gradient-corrected exchange-correlation functionals. *Theor. Chem. Acc.* **2005,** *114***,** 145–152. https://doi.org/10.1007/s00214-005-0655-y.
21. Nosé, S. A molecular dynamics method for simulations in the canonical ensemble. *Mol. Phys.* **1984**, *52* (2), 255-268. https://doi.org/10.1080/00268978400101201.
22. Nosé, S. A unified formulation of the constant temperature molecular dynamics methods. *J. Chem. Phys.* **1984**, *81*, 511-519. https://doi.org/10.1063/1.447334.
23. Hoover, W. G. Canonical dynamics: Equilibrium phase-space distributions. *Phys. Rev. A.* **1985**, *31* (3), 1695-1697. https://doi.org/10.1103/PhysRevA.31.1695.
